# Vom Protest- zum Quarantänejahr: Neue Arenen der Konfliktaushandlung

**DOI:** 10.1007/s42597-020-00047-9

**Published:** 2020-10-30

**Authors:** Tareq Sydiq

**Affiliations:** Zentrum für Konfliktforschung, Philipps-Universität Marburg, Ketzerbach 11, 35032 Marburg, Deutschland

**Keywords:** Soziale Bewegungen, Proteste, Resistance Studies, Konfliktaushandlung, Covid-19, Social movements, Protests, Resistance studies, Conflict articulation, Covid-19

## Abstract

Ob Hong Kong, Arab Spring 2.0, die Proteste in Südamerika oder im Iran, 2019 war ein Jahr globaler Protestbewegungen die insbesondere öffentliche Räume einnahmen. Manche dieser Protestbewegungen konnten schon 2019 Erfolge erzielen oder sich bereits konsolidieren; die meisten jedoch verließen sich darauf, 2020 weiter Druck aufzubauen auf Regierungen. Mit der globalen Pandemie wurde dies fast schlagartig unmöglich gemacht: Kollektive Entscheidungsgewalt, weiterhin eine Domäne der Regierungen, wurde zum entscheidenden Faktor bei der Krisenbekämpfung, auf die partikulär agierende Protestbewegungen keine unmittelbare Antwort hatten. Gleichzeitig fiel der öffentliche Raum als entscheidende Arena für die Austragung dieser Konflikte unter den Beschränkungen der Pandemie-Bekämpfung aus. Protestierende wurden damit aus dem Zentrum des öffentlichen Raumes in die Peripherie gedrängt, wo sie mittels dezentraler Techniken versuchen, Mobilisierungen aufrecht zu erhalten, und in der Pandemiebekämpfung mit staatlichen Institutionen konkurrierten. Während dies den Spielraum von Protestbewegungen einschränkt, erlaubt das Herausbilden neuer Konfliktarenen es ihnen, sich für künftige Protestbewegungen bereit zu halten und staatliche Akteure herauszufordern. Dieses Zeitfenster können Regierende nutzen, um aus effizienter Pandemiebekämpfung Kapital zu schlagen und aus der Interaktion von Institutionen mit AktivistInnen Kooperationen zu erzeugen; ohne aber die zugrundeliegenden Probleme zu lösen, vertagen sich die Protestbewegungen lediglich auf eine Zeit nach der Pandemie.

## Abstract (en)

Protest movements centered around public spaces emerged globally last year, in Hong Kong, South America, Iran or during the Arab Spring 2.0. While some could already achieve successes or consolidate themselves in 2019, most were hoping to continue pressuring governments and building momentum in 2020. The global pandemic squandered these hopes rapidly: collective decision making, still in the domain of governments, became a crucial factor in fighting the crisis, and protest movements as independent actors did not have a direct response. Simultaneously, public space as an arena for conflict articulation became inaccessible due to anti-pandemic efforts. Protestors were thus driven out of the center of public spaces and into the periphery, where they employed decentralized techniques in order to maintain their mobilization and to compete with state institutions in offering policy choices regarding anti-pandemic efforts. While limiting repertoires of protest movements, new conflict arenas allow them to sustain mobilization for future protest movements and continue challenging state actors. This period of limited protest activities can be used by governments, to accumulate political capital from efficient anti-pandemic policies and to foster cooperations with protesters. Unless they solve underlying causes of protests, however, protest movements will merely delay their activites to a time after the pandemic.

## Einleitung

Noch Anfang dieses Jahres war die Rede vom Protestjahr 2019 als einem Höhepunkt eines Jahrzehntes der Proteste (Aytac and Stokes 2020; Böge et al. 2019; Brannen and Haig 2020). Bereits kurze Zeit später, nachdem die WHO Covid-19 zu einer globalen Pandemie erklärte und weitreichende Gegenmaßnahmen von den einzelnen Staaten beschlossen wurden, scheinen diese Proteste weitgehend vergessen; zumindest sind sie aus der öffentlichen Wahrnehmung weitgehend verschwunden und finden generell eher im kleinen Rahmen statt (de Haldevang 2020). Die Pandemie, so scheint es, hat geschafft, was staatliche Repressionen nicht zu bewältigen schienen; sie hat den Protestbewegungen einen jähen Dämpfer versetzt und sie vorerst beendet. Gleichzeitig sind die den Protesten zugrunde liegenden Probleme weder gelöst, noch haben sie an Relevanz verloren; sie sind nur kurzzeitig weniger salient geworden, die Konflikte eingefroren, und wie die etwa die Proteste in den USA zeigen, können sie jederzeit wieder ausbrechen. Die Protestierenden probieren währenddessen verschiedene Alternativen aus – sie sprühen Graffitis, machen Lärm, verlagern Demonstrationen ins Internet oder in Autokorsos. Bislang scheint keiner dieser Ansätze erfolgreich das Besetzen öffentlicher Räume und den Druck auf Eliten zu ersetzen, die 2019 zu den Hauptstrategien der Protestierenden zählten. Die bewährten Strategien hatten sich im politischen Normalzustand bewährt; im Ausnahmezustand stehen die Protestierenden vor der Herausforderung, neue Strategien und Ansätze zu entwickeln, während sich die politische Arena fortlaufend wandelt.

## Konfliktaushandlung und Protestbewegungen

Soziale Ordnungen produzieren verschiedene Formen der institutionalisierten Konfliktaushandlung. Diese mögen sich mal weniger formalisiert auf gesellschaftlicher Ebene, mal stärker formalisiert in staatlicher Form manifestieren, sie sind jedoch stets grundsätzlich präsent. Die Aushandlung solcher Konflikte erfolgt ritualisiert und institutionalisiert: Repertoires, wie sie Tilly für Großbritannien identifiziert (Tilly 1993, 2008), bilden sich heraus, inspirieren ähnliche Aktionsformen und folgen ähnlichen strategischen Überlegungen. Diese Rituale finden dabei an ähnlichen, meist zentralen Räumen statt und folgen ähnlichen Mustern, wie etwa Demonstrationszügen oder Kundgebungen.

Die meisten Protestbewegungen münden nicht in einer kompletten Umwälzung gesellschaftlicher und staatlicher Verhältnisse; sogar ein Regimewechsel ist relativ selten. Die meisten Protestbewegungen setzen sich vielmehr mit kleinteiligen inhaltlichen Zielen auseinander. Ihr Erfolg bemisst sich daran, inwiefern sie auf Institutionen zu ihren Gunsten einwirken können; und Institutionen haben ein Interesse daran, sie entweder klein zu halten, oder frühzeitig Konzessionen zu gewähren um eine grundsätzliche Gefährdung der existierenden Verhältnisse zu vermeiden. Solche nach oben hin durchlässigen Institutionen sind daher nicht nur in Demokratien verankert, sondern existieren auch in Autokratien als stabilisierende Mechanismen (Marquis and Bird 2018). Umgekehrt impliziert die Existenz sozialer Institutionen auch eine Sicherheit für Demonstrierende: Wer friedlich protestiert, kann auf die öffentliche Meinung hoffen und die politischen Kosten von Repressionen erhöhen (Chenoweth and Stephan 2011). Protestierende adressieren damit verschiedene Institutionen zeitgleich, und sie lernen von vergangenen Protesten, welche Ansätze erfolgversprechender sind. Die Nutzung zentraler Plätze symbolischer Orte etwa ist oftmals stark ritualisiert, wenn Demonstrationen zum internationalen Arbeitertag zeitgleich am selben Ort stattfinden oder Forderungen über Kundgebungen und Slogans erhoben werden. Diese Ritualisierung bietet Vorteile sowohl für Regierende als auch für Protestierende: Die Berechenbarkeit verringert die Gefahr gewaltsamer Eskalationen und liefert Präzedenzfälle, um auf Institutionen einwirken zu können. Die relative Stärke von Protestbewegungen lässt sich dabei im öffentlichen Diskurs bestimmen, weswegen Teilnehmerzahlen eine wichtige Rolle spielen; nur damit lässt sich eine Messzahl wie Momentum bestimmen (Chenoweth and Belgioioso 2019).

In der Friedens- und Konfliktforschung ist insbesondere in den letzten Jahren diskutiert worden, welche konkreten Mechanismen zum Erfolg friedlicher Protestbewegungen beitragen. Omar Wasow argumentiert etwa, dass Medien eine wichtige intermediäre Institution darstellen; während der Bürgerrechtsbewegung in den USA konnten Protestierende strategisch auf die Berichterstattung einwirken, erst durch die Interaktion mit Eliten ließen sich aber Frames festsetzen (Wasow 2020). Kadivar, Usmani und Bradlow dagegen betonen die langfristigen Effekte von Protestbewegungen; die Organisation von Bewegungen nach innen erlaube auch demokratische Reformen nach außen, während Veteranen von Protesten sowohl in staatlichen Institutionen als auch in gesellschaftlichen Institutionen aufsteigen und künftige Entwicklungen beeinflussen können (Kadivar et al. 2020), ein Argument das sich so ähnlich auch bei Jonathan Pinckney findet, welcher außerdem auf die Fähigkeit Protestierender hinweist, Regierende zur Rechenschaft zu ziehen (Pinckney 2018). Auch wenn sich diese Ausführungen vor allem auf Transition beziehen, deuten sie in eine ähnliche Richtung bei der Untersuchung von Protesten: Ihre genauen Auswirkungen erschließen sich erst, wenn die Institutionen und Rahmenbedingungen berücksichtigt werden relativ zu den verwendeten Strategien der Protestierenden. Diese Rahmenbedingungen werden durch die Pandemie jedoch rasant und drastisch verändert; sowohl der öffentliche Raum, als auch die öffentliche Meinung und Institutionen reagieren auf die Pandemie und schaffen damit ein verändertes Handlungsfeld, auf das sich Protestierende einrichten müssen. Wie also gehen Protestbewegungen, die sich oftmals bereits 2019 etabliert haben, mit diesen Einschränkungen bezüglich des öffentlichen Raumes und neuen institutionellen Herausforderungen um? Wie ich im Folgenden skizzieren will, reagieren sie mit einem Rückzug in periphere Räume, während staatliche Akteure Handlungsmacht (zurück-)gewinnen. Dies eröffnet neue Handlungsräume für Kooperation und Konfliktbewältigung; solange diese aber von Regierenden nicht aktiv genutzt wird, wird diese Chance ungenutzt verstreichen.

## Das Protestjahr 2019: Revolution in Progress

Vor der Pandemie nahm die Anzahl der Protestbewegungen zu, die auf eine Mischung aus Staats- und Institutionenskepsis sowie den Gebrauch öffentlichen Raumes setzten. Dies lag sicherlich an der Frustration zuvor gescheiterter Reformbewegungen in den 2000ern und den Protesten in den 2010ern wie dem arabischen Frühling sowie den einhergehenden autoritären Antworten und Repressionen (Ambrosio 2016; Aras and Falk 2016; Battaloglu and Farasin 2017; He 2014). Das Jahr 2019 begann revolutionär: Die seit Dezember des vorherigen Jahres anhaltenden Proteste im Sudan führten erst zum Bruch von ehemaligen politischen Verbündeten mit der Regierung und mündeten schließlich zur Absetzung al-Bashirs durch das Militär am 11. April. Al-Bashir hatte zuvor nach einem Putsch 30 Jahre lang als sudanesischer Präsident regiert. Etwa zeitgleich löste die Ankündigung des seit 20 Jahren als algerischer Präsident amtierenden Bouteflika, für eine weitere Amtszeit zu kandidieren, massive Proteste aus, die zu seinem Rücktritt am 02. April führten. Außerdem begannen im März die Proteste um ein umstrittenes Auslieferungsgesetz in Hong Kong. Eine zweite Welle an Protesten begann im Oktober mit den irakischen Protesten gegen Korruption und ausländische Einflussnahme, welche zum Rücktritt des Premierministers führten. Darauf folgten die chilenischen Sozialproteste, welche ebenfalls den Rücktritt des Präsidenten und ein noch ausstehenden Verfassungsreferendum erwirkten, die libanesischen Proteste aufgrund derer der Premierminister zurücktrat, die Proteste gegen Wahlfälschung bei der bolivianischen Wahl 2019, die die Opposition an die Macht brachten, die iranischen Novemberproteste, welche brutal niedergeschlagen wurden, sowie die landesweiten Proteste in Indien gegen ein Staatsbürgerschaftsgesetz, welches muslimische Inder*innen zu benachteiligen drohte. In anderen Ländern fanden kleinere Proteste statt, sowohl in Solidarität mit anderen Ländern als auch an lokalen Problemen orientiert.

Die Proteste waren sehr unterschiedlich, sowohl was ihre inhaltliche Ausrichtung, als auch die Zusammensetzung der Protestierenden anging. Sie ähnelten sich allerdings in zwei Eigenschaften. Zum einen schienen insbesondere die Protestierenden in Algerien und im Sudan bemüht, Fehler aus dem arabischen Frühling nicht zu wiederholen, und bestehenden Institutionen nicht verfrüht zu vertrauen. Rücktritte wurden nicht akzeptiert als Lösungen; auch nachdem die Machthaber abgetreten waren, hielt die Mobilisierung an, um den Druck aufrecht zu erhalten, bis die Forderungen nicht nur symbolisch erfüllt waren. Ähnliches ließ sich auch im Libanon, in Hong Kong oder in Chile beobachten – das Erfüllen ihrer Forderungen demobilisierte die Protestierenden nicht, sondern sie bemühten sich, weiter präsent zu bleiben. Und diese Präsenz war räumlich – die andere Parallele der Protestbewegungen war ihr Bestreben, öffentliche Räume, also Plätze und Straßen, für sich zu beanspruchen. Der Deutungshoheit des Staates sollte bewusst eine Deutungshoheit der Straße entgegengesetzt werden; nur so ließe sich langfristig Druck ausüben und verhindern, dass auf den abgesetzten Alleinherrscher der nächste Alleinherrscher folgen würde.

Die sozialen Bewegungen taten denn auch, was soziale Bewegungen tun: Die bildeten eigene Organisationsformen und -strukturen heraus. Eine solche Verstetigung soll das anhaltende Wirken Protestierender sichern (McCarthy and Zald 1977; della Porta and Diani 2009, S. 150). Momentum, definiert durch die Teilnehmerzahlen und die Häufigkeit von Protestevents (Chenoweth and Belgioioso 2019), sollte aufrecht erhalten werden, während zunehmend durch Organisationen auf die öffentliche Meinung und Institutionen eingewirkt würde, gerade um zu vermeiden dass durch abnehmenden öffentlichen Druck Versprechen der Regierenden nichtig würden.

## Politik der Krise: Akteurskonstellationen und institutionelle Herausforderung

Im Dezember wurde der erste Fall von Covid-19 in China bekannt, woraus sich im Frühjahr 2020 eine globale Pandemie entwickelte. Dies ging mit großen Einschränkungen für Institutionen ebenso wie für Protestierende einher; dieses Zeitfenster nutzten Regierende jedoch mit sehr unterschiedlichem Erfolg. Mit der Pandemie entstand nun ein Politikfeld, dass sich als einziges nur kollektiv und institutionell lösen ließ – staatlich eben. Dieselben Institutionen, gegen die Protestierende vorgegangen waren, stellte damit die größte Hoffnung in der Krisenbekämpfung dar. Für die Regierenden bedeutete dies sowohl eine Chance als auch eine Herausforderung – wo sie bereits viel politisches Kapital in der Bekämpfung Protestierender verbraucht hatten, wie etwa im Iran, in welchem im November 2019 Proteste blutig niedergeschlagen wurden (Amnesty International 2019), blieb die Bevölkerung äußerst skeptisch gegenüber zentral koordinierten Maßnahmen gegen Covid-19; wo sie aber grundsätzlich Legitimität bewahrten, konnten sie nun durch eine effektive Krisenbekämpfung überzeugen. Regierungen verlassen sich in solchen Krisensituationen grundsätzlich auf zwei parallel wirkende Mechanismen, die auf politischem Kapital basieren: Eine Verankerung in der Bevölkerung erlaubt es Ihnen, zügig Gefahrenherde zu erkennen und dort aktiv zu werden, wo die Bedarfslage am größten ist, während Vertrauen in die Regierungstätigkeit dazu führt, dass auch informellen Aufforderungen und Empfehlungen der Regierenden zügig Folge geleistet wird. Beides war kurze Zeit nach den blutigen Repressionen des vergangenen Jahres sowie den Wahlen, welche im Februar 2020 noch während des ersten Ausbruchs durchgeführt wurden, im Iran nicht gegeben. Ähnlich düster sah noch im Februar die Lage in Hong Kong aus, welches ebenfalls im Februar nicht nur den Nachrichtendienst Bloomberg an einen failed state erinnerte (Clara Ferreira Marques 2020), nachdem es seit März 2019 eine langanhaltende Protestbewegung erlebt hatte, welche der amtierenden Regierung eine schwere Niederlage bei den Wahlen zufügen konnte und infolge derer die Zustimmungswerte der Regierungschefin Carrie Lam auf einen historischen Tiefstand sanken. Die Stadt schien ebenso gelähmt wie die Stadtverwaltung. In beiden Situationen erlebten die Staaten aber eine kurze Pause von ihrer Protest-Krise aufgrund der Pandemie: Massenproteste erschienen als ein Gefahrenherd, weswegen sowohl Gesellschaft als auch Staat diese einschränkten, und die Stunde der Exekutive setzte eine gewisse Zusammenarbeit mit dem Staat voraus.

In Hong Kong waren es aber vor allem gesellschaftliche Akteure und die Protestbewegung, die gegen den Virus aktiv wurden: So informierten sie über die Pandemie, Infektionsquellen, und das korrekte Tragen von Masken (trotz eines Verhüllungsverbots in der Öffentlichkeit), während sie gleichzeitig Druck auf die Regierung ausübten, die Grenzen zu China zu schließen und das Personal in Krankenhäusern besser auszustatten (Tufekci 2020). Im Iran unterstützten Freiwillige die Regierung bei solchen Maßnahmen, unter anderem indem sie Orte desinfizierten und Spenden sammelten (Basravi 2020). Während die Regierung noch im Februar die Krise herunterredete, da sie die Wahlen am 21.02.2020 nicht gefährden wollte, verbreiteten Kanäle auf sozialen Medien und Messengern wie Telegram bereits Informationen und spekulierten über den wahren Stand der Infektionen, etwa in den Gefängnissen (Amini 2020). Aktivist*innen verlagerten in beiden Fällen den Fokus weg von ihren unmittelbaren politischen Interessen hin zur Pandemiebekämpfung. Der Druck auf die Regierung, effizienter gegen die Pandemie vorzugehen, brachte sie weiterhin in eine Konfrontation mit der Regierung, nun jedoch in einem neuen Politikfeld. Ihre Informationskampagnen ergänzten aber auch Maßnahmen staatlicher Institutionen zur Pandemiebekämpfung oder führten bei Nachbarschaftshilfen sogar zu (begrenzten) faktischen Kooperationen. Diese Verlagerung war der veränderten Situation geschuldet, nicht einer grundsätzlichen Interessensverlagerung; sie erlaubten es Protestierenden, ihre organisationalen Kapazitäten zu bewahren, indem sie ein neues Aufgabenfeld entdeckten, anstatt aufgrund von Inaktivität zu Demobilisieren. Die Herausforderung staatlicher Institutionen in einem neuen Politikfeld verlieh ihnen zusätzliche Legitimität und erlaubte es, den Einfluss staatlicher Behörden einzuschränken.

Die Stunde der Exekutive in der Krise, in welcher Hilfsgelder fließen und auf Regierende geschaut wird, führte zu zahlreichen Fällen von Korruptionsvorwürfen und Machtkonsolidierung – ersteres etwa in Brasilien, den USA und Kenia, letzteres unter anderem in Ungarn und auf den Philippinen (Jarvis 2020). In Israel beschleunigte die Krise die Bildung einer Einheitsregierung und trug damit zur Spaltung der größten Oppositionspartei bei. Währenddessen bauten autoritäre Herrscher, wie al-Sisi in Ägypten, ihre Befugnisse unter Einsatz von Notstandsgesetzen und einer Politik der Krise aus (Transparency International 2020). Die Krise bremste dadurch das Momentum und die Dynamik von Protestbewegungen, während die Regierenden ein Fenster öffnete, sich zu konsolidieren. Dieses Bremsen drängte die Bewegungen aber nicht zurück, es fror sie lediglich auf dem Stand vor der Krise ein, solange sie durch veränderte Aktivitäten eine Demobilisierung verhindern konnten. Wie dieses Fenster genutzt wurde, unterscheidet sich stark von Land zu Land, und eine zügige, effektive Pandemiebekämpfung kann Regierenden zusätzliches politisches Kapital verschaffen, während eine zögerliche Reaktion lokale Entscheidungsträger in den Vordergrund drängen lässt (Blofied et al. 2020). Während nun eine Pandemie historischen Ausmaßes einen großen Teil der begrenzten Ressource „Medienaufmerksamkeit“ einnahm und Regierungsaktivitäten der Pandemiebekämpfung sowohl den öffentlichen Diskurs, als auch den öffentlichen Raum für sich einnehmen konnten, verdrängten diese Dynamiken Protestierende in die Peripherie. Gerade für Regierungen, die durch Protestbewegungen 2019 in die Krise geraten waren, eröffnete sich hier eine Chance, verlorenes Vertrauen wieder herzustellen durch effektives Krisenmanagement und zusätzliche Zeit zu gewinnen bei der Lösung zugrundeliegender Konfliktlagen. Wie ich im folgenden Kapitel ausführen will, ist dies allerdings weitgehend ausgeblieben; stattdessen forderten die Protestbewegungen ineffiziente Verwaltungen weiterhin heraus, während unveränderte Konfliktlagen nur darauf warten, nach der Pandemie wieder in Protestbewegungen aus der Peripherie heraus auszubrechen.

## Neue Arenen der Konfliktaushandlung

Während als die Pandemie Rahmenbedingungen schaffte, in denen Protestierenden sowohl der öffentliche Raum als auch Aufmerksamkeit für ihre Themen verwehrt blieben, standen sie vor der Herausforderung, ihre neue Handlungsräume zu eröffnen. Dies taten sie, indem sie neue Arenen der Konfliktaushandlung eröffneten – neben den neuen Politikfeldern betraf dies die Orte, an denen diese Politikfelder ausgehandelt wurden. Trotz dieser Einschränkungen und der Ausweitung staatlichen Handelns verschwanden Protestbewegungen nämlich nicht. Ihre Aktivitäten passten sich lediglich an und arrangierten sich mit den Auflagen bezüglich öffentlicher Proteste. Sogar im von der Pandemie als erstes getroffenen Wuhan fanden daher Proteste statt, etwa von Feministinnen. Honwei Bao beschreibt den Kontext dazu folgendermaßen: „The lockdown condition offers ample opportunities for activist campaigns because many people – mostly the ‚non-essential‘ workers in the public policy discourse – now have more time and enthusiasm for social participation.“ (Bao 2020) Die von Bao beschriebenen Proteste finden dabei insbesondere im öffentlichen Raum statt, wenn auf schwarzen Brettern offene Briefe angebracht werden und dieser Raum damit von Aktivistinnen in Anspruch genommen wird (Bao 2020). Dieser kreative Nutzen von öffentlichem Raum war auch in Deutschland zu beobachten, beispielsweise durch Graffitis, welche einzelne Spaziergänger während der Kontaktbeschränkungen erreichen sollten. Der öffentliche Raum war nicht wie gewohnt durch breite Gruppen zugänglich, wurde aber so durch einzelne Akteure temporär markiert; insbesondere durch die Interaktion mit dem *built environment*. Andere gingen einen Schritt weiter – in Israel protestierten Autokorsos gegen dem amtierenden Regierungspräsidenten Netanjahu aufgrund von Korruptionsvorwürfen (Hasson et al. 2020).

An anderen Orten wurde mit Geräuschen Raum eingenommen. In Teheran etwa sangen Anwohner verbotene Lieder von ihren Balkonen, eine gleich doppelte Provokation in einem Land, welches öffentliche Musik einschränkt und weitgehend verbietet (Yaghoub Fazeli 2020). In Brasilien schlugen Protestierende in sogenannten *panelaços* auf Töpfe, um ihrer Wut Ausdruck zu verleihen (Fischermann 2020). Anders als durch Demonstrationen sind diese Proteste nicht auf zentralen Plätzen sichtbar; sie sind aber dezentral, im Umkreis der Lautquelle, wahrnehmbar.

Wo physischer Protest nicht möglich ist, bietet das Internet einen Ausweg. In digitalen Resonanzräumen lässt sich Unmut kund tun, aber auch demonstrativ organisieren; knapp 20.000 partizipierten am Livestream von Fridays for Future (Tagesschau 2020). In Singapur erzeugte das verlagern von Protestaktivitäten aus der Offline- in die Online-Welt dazu, dass Fragen zur strategischen Ausrichtung offener diskutiert werden konnten (Ng Yu Ling 2020). Diese Formen von Aktivismus dürften aber stärker nach innen als nach außen wirken; während sie Debatten über die eigene Ausrichtung beziehungsweise Strategien befördern und eine Sichtbarkeit von Solidarität erzeugen, blieb die Medienresonanz begrenzt. Die Wirkung auf öffentliche Debatten, die von Massenprotesten ausgehen kann, blieb weitestgehend aus; stattdessen könnten Online-Proteste Bewegungen aufrecht erhalten für eine Zeit nach Corona, indem sie die Einheit und Motivation von Bewegungen nach innen signalisieren. Dass diese Online-Proteste dennoch Regierende beunruhigen können, wurde offenbar, als im Iran der Hashtag „nicht hinrichten“ trendete. In Millionen Beiträgen auf Instagram und Twitter nach der Verurteilung dreier Studenten wegen ihrer Teilnahme an den Novemberprotesten zum Tode; das Oberste Gericht des Irans ließ daraufhin eine Wiederaufnahme des Verfahrens zu (Malekian 2020).

Besonders entfalten konnten sich solche Online-Proteste aber erst da, wo der Frust über staatliches Versagen zu einer Verschränkung physischer und digitaler Proteste führte. Diese waren gleichzeitig das Ergebnis einer so tiefgehenden Staatsskepsis, dass auch Sicherheitsmaßnahmen zur Pandemie sie nicht unterbinden konnten. Bei den Protesten anlässlich der Tötung George Floyds durch US-amerikanische Polizisten etwa verschärften die häufig drastischen Antworten der Sicherheitskräfte die Konflikte enorm, eine Kooperation schien weitgehend unmöglich. Eine besonders markante Demonstration dieser Skepsis gegenüber dem Staat dürfte die Capitol Hill Autonomous Zone (CHAZ) in Seattle darstellen, in welcher die Polizei explizit unerwünscht ist. Solche autonomen Zonen sind nichts neues, bereits in den 90ern wurde der Gedanke, staatsfreie Räume zumindest temporär zu schaffen, in anarchistischen Kreisen breit diskutiert und umgesetzt (Bey 2008); die Skepsis gegenüber dem Staat und insbesondere seinem repressiven Apparat schien hier aber nicht nur auf Anarchisten und Autonome Aktivisten beschränkt, sondern in weiten Teilen der Gesellschaft verankert und durch die Proteste befördert. Flankiert wurden diese Proteste mit Online-Aktionen, die dezentrale Protestformen koordinierten, informierten und sogar die Arbeit von Sicherheitsbehörden erschwerten indem sie etwa deren Aufenthaltsort öffentlich machten^1^. Im Umgang mit der Pandemie entwickelten Protestierende schließlich eigene Strategien; Maskenpflicht, Distanzierung und Desinfektionen wurden zu gängigen Mitteln, Empfehlungen supranationaler Akteure wie der WHO nahmen einen größeren Rahmen ein als Empfehlungen der eigenen Regierung.

## Herausforderungen und Ausblick

Während der Pandemie eröffneten sich Handlungsräume für Regierende; diese wurden jedoch selten dazu genutzt, zugrundeliegende Konfliktlagen zu lösen. Stattdessen erzeugte die Dominanz staatlicher Institutionen auf dem Politikfeld der Pandemiebekämpfung und in zentralen öffentlichen Räumen eine Verdrängung der Protestierenden. Diese erarbeiteten sich neue Arenen der Konfliktaushandlung: Dezentral, nicht-körperlich und digital. Essentiell für den Verlauf von Protestbewegungen während er Pandemie scheint dabei die Fähigkeit Regierender, durch ein effektives Krisenmanagement Vertrauen herzustellen, sowie die Fähigkeit Protestierender, auf Fehler hinzuweisen und dieses Vertrauen zu verringern.

Den genannten Protesten ist gemeinsam, dass sie durch ihre dezentrale, unpersönliche Art eine Vernetzung und Verstetigung ad-hoc und vor Ort erschweren. Eine befreundete Aktivistin aus den USA zeigte sich frustriert über die Black-Lives-Matter-Bewegung, da sie wegen Corona nicht wisse, was sie anderes tun solle als protestieren; es fehle eine Perspektive für den darauffolgenden Schritt. Einige Politikziele, wie „Defund Police“ wurden zwar formuliert und ihnen wurde auf Protesten Raum gegeben; es bleibt aber offen, inwiefern diese sich durch Organisationen auch in konkrete Politik übersetzen lassen. Ähnlich frustriert zeigen sich Aktivist*innen aus dem Iran – die Revolution scheint vorerst auszubleiben, selbst kleinste Erfolge wie die Neuverhandlung der Todesurteile dreier Protestierender sind bemerkenswert. Zwar erzeugt die Krise ein gemeinsames Politikfeld und dadurch eine Chance für Kooperationen und Aussöhnungen; diese sind aber kein selbstverständliches Ergebnis, sondern müssten aktiv befördert werden, wie es etwa Nadine Ansorg und Julia Strasheim in der Friedensarbeit fordern (Ansorg and Strasheim 2020). Solange aber weder Konzessionen gewährt noch die Konfliktlinien, welche die ursprünglichen Proteste angestoßen haben, überwunden werden, dürften die Proteste wiederkehren.

In der Nachbarschaftspolitik und im Internet folgen diese Konflikte einem anderen Drehbuch als auf der Straße. In konventionellen Protesten erzeugt öffentlicher Druck Medienaufmerksamkeit und Druck auf Behörden, welche Politiken übernehmen oder sich mit gebildeten Organisationen sozialer Bewegungen oder bewegungsnahen Akteuren auf Verhandlungen einlassen. Das Ausfallen öffentlicher Resonanzräume für Proteste erzeugt dagegen einen Innovationsdruck auf Protestierende, welcher in autoritären Staaten aufgrund repressiver Politiken allerdings bereits seit langem bestand und zu kreativem Nutzen öffentlichen Raumes führte (Sydiq 2020). Dezentrale Proteste sind wesentlich weniger stark repressiven Apparaten ausgesetzt, sie sind aber auch weniger sichtbar außerhalb der jeweiligen Blasen und erschweren spontane Allianzbildungen über diese Blasen hinaus; während sie lange überdauern können, fällt es ihnen schwer, zunehmend steigenden Druck auszuüben, der schließlich in Erfolgen mündet. In Form von Nachbarschaftshilfen und alternativen Kommunikationsplattformen lösen Protestierende schließlich Probleme, derer staatliche Institutionen nicht Herr werden; sie fordern damit staatliche Institutionen indirekt in ihrer Hoheit über diese Politikfelder heraus und bilden Gemeinschaften, die sowohl ideell als auch sozial vernetzt sind. Solange Regierende sich dieser nicht annehmen, dürften aus diesen heraus neue Proteste ausbrechen, solange entweder das Pandemierisiko ausreichend absinkt oder die Frustration über Regierende ausreichend zunimmt. Kooperationen in der Pandemiebekämpfungen eröffneten hierzu vielversprechende Möglichkeiten; bislang sieht es aber so aus, als würden diese ungenutzt verstreichen.

## Footnotes

1 In einem Projekt wurde Polizeikommunikation als Karte dargestellt, was Aufschluss über deren Einsatzort gibt: https://scanmap.mobi. Andere Projekte anonymisieren Fotos automatisiert (https://everestpipkin.github.io/image-scrubber/) oder fluteten Webseiten und Apps der Sicherheitsbehörden mit Informationen, bis diese überfordert wurden.
